# *Aspergillus oryzae* solid-state fermentation enriches protopanaxatriol-type ginsenosides in *Panax ginseng* and confers cytoprotective effects *in vitro*

**DOI:** 10.3389/fmicb.2026.1747324

**Published:** 2026-03-06

**Authors:** Xinyang Li, Hu Ding, Ergang Wang, Shumin Wang, Huan Wang, Changbao Chen

**Affiliations:** 1 Jilin Ginseng Academy, Changchun University of Chinese Medicine, Changchun, China; 2 College of Pharmacy, Changchun University of Chinese Medicine, Changchun, China

**Keywords:** *Aspergillus oryzae*, biotransformation, GES-1, ginsenosides, α-L-arabinopyranosidic

## Abstract

**Background:**

This study established and optimized a food-grade solid-state fermentation (SSF) process using *Aspergillus oryzae* to biotransform ginsenosides in five-year-old white ginseng roots.

**Methods:**

Through single-factor and orthogonal tests, optimal SSF conditions were identified. UPLC-QTOF-MS/MS analysis was used to characterize ginsenoside profile changes. In an ethanol-induced injury model using GES-1 gastric epithelial cells, the fermented extract was evaluated for cytoprotective effects.

**Results:**

Optimal SSF conditions were fermentation time of 8 days, inoculum size of 2.5%, and temperature of 28°C. UPLC-QTOF-MS/MS analysis revealed significant remodeling of protopanaxatriol (PPT)-type ginsenosides, with ginsenoside Rf and PPT increasing by 3.55-fold and 5.03-fold, respectively (*p* < 0.05). In the ethanol-induced injury model using GES-1 gastric epithelial cells, the fermented extract demonstrated dose-dependent anti-inflammatory, antioxidant, and anti-apoptotic effects without cytotoxicity. We hypothesize that the extracellular glycosidase activity of A. oryzae mediates the sequential deglycosylation leading to the observed PPT-type enrichment, although the specific enzymes involved require further identification. Overall, these results provide a proof-of-concept for a food-safe SSF platform tailored to whole white ginseng roots. This process effectively remodels the ginsenoside profile to enrich cytoprotective PPT-type compounds, supporting its potential for nutraceutical development.

## 1 Introduction

*Panax ginseng* C. A. Mey. is widely used as a raw material for health foods and nutraceuticals, and its pharmacological properties are largely attributed to ginsenosides (Lee et al., 2019; Ratan et al., 2021). The predominant prototype ginsenosides (e.g., Rb1, Re, Rg1) exhibit limited oral bioavailability because of their high molecular weight and polarity, which constrains their clinical and functional-food effectiveness (Liu et al., 2015). Conversion of these abundant prototypes into rarer, less glycosylated ginsenosides (for example, PPT, Rh1, Rh2) has therefore become an important strategy, since these derivatives often show improved absorption and pronounced bioactivities relevant to neuroprotection, anti-inflammation, and gastroprotection (Zhang et al., 2017; Lee et al., 2021; Choi et al., 2011; Jung et al., 2010; Yu et al., 2020).

Methods for generating rare ginsenosides encompass chemical, physical, enzymatic, and microbial approaches, each presenting distinct trade-offs. Chemical hydrolysis is straightforward but often lacks regioselectivity, leading to undesired byproducts (Shin and Oh, 2016). Physical methods such as ultrasound-, microwave-, or high-pressure-assisted processing can enhance extraction efficiency and sometimes facilitate structural modifications, yet they may require precise energy input control and often serve as auxiliary steps rather than standalone conversion pathways (Ji, 2013; Zhao, 2019). In contrast, enzymatic catalysis using purified enzymes offers high specificity under mild conditions but is frequently hampered by high costs and the complexity of orchestrating multi-step conversions (Shin and Oh, 2016; Choi et al., 2024). Microbial fermentation, particularly solid-state fermentation (SSF), presents an integrated biological alternative. SSF leverages the intact plant matrix, stimulates high titers of secreted hydrolytic enzymes, and can be more resource-efficient than submerged fermentation, making it a promising route for direct biotransformation within the raw material (Thomas et al., 2013; Patel et al., 2022).

Choice of microorganism strongly influences transformation specificity (Xu et al., 2015). Many studies have focused on fungi such as *Aspergillus niger*, which efficiently hydrolyzes protopanaxadiol (PPD)-type ginsenosides (e.g., Rb1 to Rg3) but shows limited activity toward protopanaxatriol (PPT)-type compounds (Wang et al., 2011; Chang et al., 2014). In contrast, the food-grade fungus *Aspergillus oryzae* possesses a diverse enzymatic repertoire and has been reported to exhibit a preferential ability to transform PPT-type ginsenosides, suggesting a distinct biocatalytic profile suited for enriching this valuable fraction (Wang et al., 2011; Noh et al., 2017; Uwineza et al., 2024; Yang et al., 2024). Its GRAS (generally recognized as safe) status further makes it an ideal candidate for producing nutraceutical ingredients. However, a systematic approach that combines the optimization of an *A. oryzae*-based SSF process for white ginseng, detailed tracking of the resulting PPT-type ginsenosides enrichment, and linkage of this specific chemical remodeling to enhanced biological activity, remains unexplored.

To address this gap, we developed a food-grade SSF process using *A. oryzae* applied to white *P. ginseng* with three interlinked objectives: (1) to optimize key SSF parameters (including fermentation time, inoculum size, and temperature) using single-factor tests and orthogonal test designs to define reproducible operational windows; (2) to characterize ginsenoside transformations by UPLC-QTOF MS/MS with emphasis on PPT-type conversions and quantification of marker compounds; and (3) to couple compositional changes with preliminary biological assessment, evaluating cytotoxicity and gastroprotective potential in an ethanol-induced GES-1 cell model. By combining statistically guided process optimization, high-resolution chemical profiling, and targeted *in vitro* bioassays, this study provides a reproducible SSF framework for selective enrichment of PPT-type ginsenosides and generates testable hypotheses about the enzymatic steps (for example, the conversion of Rf to PPT) for follow-up mechanistic and safety studies.

## 2 Materials and methods

### 2.1 Materials

*A. oryzae* was obtained from the China Center for Type Culture Collection (CCTCC) under strain number AF 2018017. 5-year-old white ginseng roots were purchased from Northeast Specialty Co., Ltd (Jilin, China). All components for preparing the Potato Dextrose Agar (PDA) medium-including glucose, yeast extract, agar powder (excluded in liquid PDA formulation), and potato extract powder, and ginsenosides reference standards used in this study, were sourced from Shanghai Yuanye Bio-Technology Co., Ltd (Shanghai, China). Chemicals for the preparation of fermentation extracts, including n-butanol, acetonitrile, formic acid, and methanol, were supplied by Thermo Fisher Scientific (Massachusetts, USA). The GES-1 cell line was sourced from Shanghai Fuheng Biotechnology Co., Ltd. (Shanghai, China); antibodies against Bcl-2, Bax, Nrf2, β-Actin loading control protein and horseradish peroxidase (HRP)-conjugated goat anti-rabbit and goat anti-mouse secondary antibody were sourced from Proteintech Group, Inc (Wuhan, China). Enzyme-linked immunosorbent assay (ELISA) kits for interleukin (IL)-8, IL-10, IL-1β, tumor necrosis factor (TNF)-α, superoxide dismutase (SOD1), malondialdehyde (MDA), nitric oxide (NO), and glutathione peroxidase (GSH-Px) were acquired from Jiangsu Enzyme Immunity Industrial Co., Ltd (Jiangsu, China).

### 2.2 Activation of *A. oryzae* strain and preparation of *A. oryzae*-ginseng solid-state fermentation substrates

The lyophilized *A. oryzae* strain (CCTCC No. AF 2018017) was revived in sterile Potato Dextrose Agar (PDA) liquid medium and incubated at 28 °C with shaking at 140 rpm for 24 h in dark. Following mycelial growth, the culture was subcultured twice on PDA plates to ensure strain purity and activity. A spore suspension was prepared by harvesting spores from the fresh mycelia with sterile 0.05% Tween 80 solution. Spore concentration was determined using a hemocytometer and adjusted to 5.7 × 10^6^ spores/mL with sterile deionized water, which served as the inoculum. The *A. oryzae*-ginseng fermentation substrate was prepared following a modified method from Tang et al. (2023). White *P. ginseng* roots were dried, ground, and sieved through a 16-mesh sieve (particle size ≤ 1 mm) to ensure uniform substrate texture and enhance microbial accessibility. For each fermentation unit, 25.0 g of ginseng powder was weighed into a sterile container. The moisture content of the substrate was adjusted to 60% (w/w) with sterile deionized water, and the substrate was then sterilized by autoclaving at 121 °C for 30 min. After cooling to room temperature in a laminar-flow hood, the experimental units were inoculated with the spore suspension according to the designed inoculum size (v/w). Control units received an equivalent volume of sterile water. All units were incubated statically at the specified temperature (e.g., 28 °C) in an environment with high relative humidity (> 90%), maintained by placing open vessels inside a larger container with moistened sterile gauze. Fungal growth and substrate morphology were visually monitored every 24 h. Each treatment was performed with five biological replicates (*n* = 5).

### 2.3 Single-factor tests

To systematically optimize the solid-state fermentation (SSF) process, a two-stage experimental strategy was employed. First, single-factor tests were conducted to identify the approximate appropriate ranges for three critical parameters: fermentation time (A), inoculum size (B), and temperature (C). The total ginsenoside content and total sugar content of the fermentation substrate were used as the primary response indicators. Subsequently, based on the results of these preliminary tests, an orthogonal array design [L_16_(4^3^)] was implemented to efficiently evaluate the interactions among these factors and determine their optimal combination. In the single-factor tests stage, each parameter was varied while the other two were held constant at a baseline level, and all tests were performed with five biological replicates (*n* = 5).

(1) Fermentation time: With the fermentation temperature maintained at 28 °C and inoculum size at 2.5%, fermentation was conducted for 2, 4, 6, 8, and 10 days.
(2) Inoculum size: With the fermentation temperature maintained at 28 °C and fermentation duration fixed at 8 days, inoculum size of 1, 2.5, 5, 7.5, and 10% (v/w) were tested.
(3) Fermentation temperature: With the fermentation duration fixed at 10 days and inoculum size at 2.5%, temperatures of 25, 28, 31, 34, and 37° were evaluated.

The total ginsenoside and total sugar contents were quantified according to the methods described by Leng et al. (2022). Results are expressed as mean ± standard deviation (SD). The experimental layout for the single-factor tests is summarized in Table 1.

**TABLE 1:** Experimental design for single-factor tests and the orthogonal test.

Stage and design	Factor	Symbol	Tested levels	Note
A. Single-factor tests	Fermentation time (d)	A	2, 4, 6, 8, 10	Other factors remain constant
	Inoculum size (%)	B	1, 2.5, 5, 7.5, 10	
	Temperature (°C)	C	25, 28, 31, 34, 37	
B. Orthogonal test	Fermentation time (d)	A	4, 6, 8, 10	The level is determined based on the trend observed in the single-factor tests section
	Inoculum size (%)	B	1, 2.5, 5, 7.5	
	Temperature (°C)	C	28, 31, 34, 37	

### 2.4 Orthogonal experimental design

Based on the preliminary ranges identified in the single-factor tests, an orthogonal array design [Taguchi L_16_(4^3^)] was employed to optimize the three key parameters at four levels each: fermentation time (A), inoculum size (B), and fermentation temperature (C). The four levels for each factor (detailed in Table 1) were selected according to the response trends observed in the single-factor experiments. The optimal fermentation conditions were determined by evaluating the total ginsenoside and total sugar contents in the fermented products under each experimental run, followed by an analysis of means and range (R) values derived from the orthogonal array. Each treatment combination was performed with five biological replicates (n = 5), and results are presented as mean ± SD.

### 2.5 Preparation of LC-MS samples

The fermented samples were extracted following a previously established method (Luo et al., 2024) with modifications. Briefly, 5.00 g (dry weight) of each sample was extracted with 100 mL of 70% (v/v) aqueous methanol (a solvent-to-sample ratio of 20:1, v/w) using an ultrasonic bath (480 W). The extraction was performed three times for 30 min each at 60 °C, with the sample allowed to cool to room temperature between extractions. After each extraction, the mixture was centrifuged at 12,000 × g for 10 min at room temperature. The resulting supernatants from the three extractions were combined and concentrated to dryness under reduced pressure using a rotary evaporator (water bath temperature set at 40 °C). The dried extract was reconstituted in 2.00 mL of 70% methanol, vortexed thoroughly, and then centrifuged at 12,000 × g for 5 min at 4 °C to precipitate insoluble particles. The supernatant was finally filtered through a 0.22 μm LC-MS compatible syringe filter. All filtered extracts were stored in amber vials at −20 °C and analyzed within 48 h. Each sample was extracted with at least five technical replicates.

Twenty-one ginsenoside reference standards were used for quantification. Individual stock solutions (1 mg/mL) were prepared by dissolving 1.0 mg of each standard in 1.0 mL of 70% (v/v) LC-MS grade methanol. These stock solutions were then separately diluted with the same solvent to prepare working solutions at concentrations of 1, 10, 20, 30, and 40 μg/mL. Mixed standard solutions for calibration were prepared by combining appropriate volumes of the individual working solutions to achieve final concentrations of 1, 10, 20, 30, and 40 μg/mL for each ginsenoside. A separate 10 μg/mL mixed standard solution was designated as the Quality Control (QC) sample. All mixed standard solutions were filtered through a 0.22 μm organic solvent-compatible filter and stored at −20 °C prior to analysis. Quantification was performed using the internal standard method. The QC sample was injected five times at random intervals throughout the analytical sequence to monitor the stability and performance of the LC-MS/MS system.

### 2.6 UPLC-QTOF-MS conditions

Chromatographic separation was carried out on an ACQUITY UPLC BEH C_18_ column (2.1 × 100 mm, 1.7 μm) maintained at 35 °C. The mobile phase consisted of A: 0.1% (v/v) formic acid in water and B: 0.1% (v/v) formic acid in acetonitrile, delivered at a flow rate of 0.3 mL/min. The injection volume was 3 μL with the sample compartment temperature set at 4 °C. The gradient elution program is detailed in Supplementary Table 1.

Mass spectrometry parameters were configured as follows: electrospray ionization (ESI) source operated in negative ion mode; MS^*E*^ acquisition mode; precursor ion m/z 554.2620 [M-H]^–^; nitrogen as nebulizing and cone gas (cone gas flow: 50 L/h, desolvation gas flow: 800 L/h); source temperatures set to desolvation 450 °C and capillary 120 °C; voltages applied at cone 40 V and capillary −3.0 kV; scan duration 0.3 s with 0.02 s interscan delay over m/z 100–1,500. Leucine-enkephalin solution (200 pg/μL) was infused at 10 μL/min as a lock mass reference for real-time calibration.

### 2.7 Cell culture and viability assay

After successful resuscitation, GES-1 cells were transferred to a 37 °C, 5% CO_2_ cell culture incubator and maintained with medium replacement every 48 h.

Cell viability was assessed using the CCK-8 assay. Logarithmic-phase GES-1 cells were seeded in 96-well plates at a density of 1 × 10^4^ cells per well and allowed to adhere. Cells were divided into four experimental groups: (1) Blank control (DMEM medium only), (2) Model group (ethanol-containing medium at 1%–7% concentrations), (3) Positive control groups (Weilexin group: 200 μg/mL, Omeprazole group: 200 μg/mL), and (4) ginsenosides extract groups (100, 200, 400, 800, 1,600 μg/mL). After 24-h incubation, CCK-8 reagent was added and incubated for 2 h. Absorbance was measured at 450 nm using a microplate reader. The optimal ethanol concentration, exposure duration, positive control groups, and ginsenosides extract concentrations for subsequent mechanistic investigations were determined through computational analysis using the established formula.


CellSurvivalRate(%)=[(As-Ab)÷(Ac-Ab)]×100%

As: Combination-intervention wells (containing cells, DMEM medium, positive control drugs, and CCK-8 reagent);

Ab: Blank control wells (containing DMEM medium and CCK-8 reagent);

Ac: Control wells (containing cells, DMEM medium, and CCK-8 reagent).

### 2.8 Anti-inflammatory and antioxidant activity

The levels of inflammatory cytokines (IL-1β, IL-8, IL-10, TNF-α) and oxidative stress indicators (SOD1, NO, MDA, GSH-Px) in the supernatants of GES-1 cells were determined using ELISA kits according to the manufacturer’s instructions.

### 2.9 Western blot

The expression of Bax, Bcl-2, and Nrf2 proteins was detected by Western blotting as previously described (Wang et al., 2024). Briefly, total protein was extracted from GES-1 cells using RIPA lysis buffer (Beyotime, P0013B) supplemented with protease and phosphatase inhibitors (Thermo Scientific, 78440) on ice for 30 min, followed by centrifugation at 12,000 × *g* for 15 min at 4 °C. Protein concentrations were determined using a BCA assay kit (Proteintech, PK10026). Equal amounts of protein (20 μg per lane) were separated by 10% SDS-PAGE and transferred onto PVDF membranes (Beyotime, FFP39) using a wet transfer system at a constant current of 120 mA for 120 min. The membranes were blocked with 5% non-fat milk for 2 h at room temperature and then incubated overnight at 4 °C with the following primary antibodies diluted in 5% BSA: anti-Bax (Proteintech, 50599-2-lg; 1:1,000), anti-Bcl-2 (Proteintech, 68103-1-lg; 1:1,000), anti-Nrf2 (Proteintech, 16396-1-AP; 1:1,000), and anti-β-actin (Mouse monoclonal, Proteintech, 60008-1-lg; 1:5,000). After washing three times with TBST, the membranes were incubated with HRP-conjugated goat anti-rabbit (Proteintech, RGAR001; 1:10,000) and goat anti-mouse (Proteintech, RGAM011; 1:10,000) secondary antibodies for 2 h at room temperature. Protein bands were visualized using an ECL substrate (Beyotime, P0018S).

### 2.10 Statistical analysis

All experimental data were organized using Microsoft Excel. Statistical analyses were performed using SPSS Statistics 22.0. For liquid chromatography-mass spectrometry (LC-MS) analysis, data acquisition and processing were conducted with MassLynx V4.2 software. Protein expression levels were quantified from Western blot bands using ImageJ software. Continuous data are expressed as the mean ± SD. Differences with a *p* < 0.05 were considered statistically significant. All figures were constructed using GraphPad Prism 9.5 and Origin 2021 software.

## 3 Results

### 3.1 Analysis of single-factor tests

The effects of fermentation duration, temperature, and inoculum size on total sugar and ginsenoside content during the solid-state fermentation of ginseng by *A. oryzae* are presented in Table 2. As shown in Supplementary Figures 1A,B, fermentation duration significantly influenced the process outcomes. Total sugar content exhibited a time-dependent increase, with the fermented group showing significantly higher levels (*p* < 0.05) than the control from day 6, peaking at 2.482 ± 0.002 mg/mL on day 10. In contrast, the total ginsenoside content in the fermented group surpassed that of the control after day 6, reaching a maximum on day 8 with a statistically significant difference (*p* < 0.05), after which it declined. Based on the optimal ginsenoside yield, an 8-day fermentation period was selected for subsequent experiments.

**TABLE 2:** Results of single-factor tests: variation of total ginsenoside and sugar contents.

Factors	Level	Experimental group		Control group	
		Total ginsenosides content (mg/mL)	Total sugar content (mg/mL)	Total ginsenosides content (mg/mL)	Total sugar content (mg/mL)
Fermentation time (d)	2	0.103 ± 0.009	0.734 ± 0.064	0.104 ± 0.003	0.780 ± 0.071
	4	0.108 ± 0.009	1.041 ± 0.068	0.110 ± 0.008	1.201 ± 0.087
	6	0.115 ± 0.008	1.510 ± 0.071	0.112 ± 0.003	1.403 ± 0.079
	8	0.122 ± 0.006	1.821 ± 0.073	0.104 ± 0.001	1.713 ± 0.088
	10	0.105 ± 0.002	2.236 ± 0.059	0.101 ± 0.004	2.016 ± 0.081
Inoculum size (%)	1	0.094 ± 0.003	2.687 ± 0.032	0.089 ± 0.010	3.179 ± 0.087
	2.5	0.127 ± 0.004	3.167 ± 0.061		
	5	0.099 ± 0.005	2.726 ± 0.072		
	7.5	0.094 ± 0.003	2.356 ± 0.022		
	10	0.092 ± 0.005	2.503 ± 0.011		
Temperature (°C)	25	0.098 ± 0.009	2.142 ± 0.103	0.086 ± 0.007	2.800 ± 0.169
	28	0.121 ± 0.007	2.988 ± 0.077	0.087 ± 0.005	2.913 ± 0.050
	31	0.105 ± 0.013	2.419 ± 0.167	0.086 ± 0.007	2.785 ± 0.160
	34	0.092 ± 0.010	2.134 ± 0.137	0.083 ± 0.002	2.807 ± 0.014
	37	0.079 ± 0.002	2.081 ± 0.106	0.080 ± 0.004	2.796 ± 0.028

The fermentation temperature was also a critical factor (Supplementary Figures 1C,D). The highest levels of both total sugar (2.958 ± 0.067 mg/mL) and total ginsenosides (1.125 ± 0.004 mg/mL) in the fermented group were achieved at 28 °C, with the ginsenoside content being significantly higher (*p* < 0.05) than in the control. Temperatures deviating from this optimum resulted in reduced accumulation of both components. Across all temperatures, the control group consistently exhibited higher sugar but lower ginsenoside levels, highlighting the essential role of *A. oryzae* metabolism in the bioconversion process. Therefore, 28 °C was established as the optimal temperature.

Finally, the influence of inoculum size was investigated (Supplementary Figures 1E,F). The optimal inoculation rate was found to be 2.5%, at which point the fermented group reached peak total sugar (3.119 ± 0.008 mg/mL) and ginsenoside (1.117 ± 0.006 mg/mL) contents, the latter being significantly higher (*p* < 0.05) than the control. An inoculum size exceeding 2.5% likely induced oxygen limitation, favoring saccharification over ginsenoside biosynthesis, while a smaller inoculum reduced fermentation efficiency. Thus, a 2.5% inoculum was determined to be optimal.

### 3.2 Analysis of orthogonal test

Based on the range (R) values from the orthogonal analysis presented in Table 3, the factors influencing the total sugar content and total ginsenosides content in the solid-state fermentation of *A. oryzae* with ginseng are ranked in descending order of significance as follows: A > B > C. This indicates that fermentation time exerted the most significant effect on both total sugar and total ginsenosides content during the process, followed by inoculation volume. In contrast, fermentation temperature exhibited a relatively minor influence. Considering all factors, the total sugar content, total ginsenosides content, and the k values, the optimal combination for the *A. oryzae*-ginseng solid-state fermentation was determined to be A2B2C3. This corresponds to fermentation conditions of 8 days, a 2.5% inoculation volume, and 28 °C, under which the fermentation performance was optimal (Supplementary Figures 1G–I). Verification experiments conducted under these optimized conditions employed three replicates per group, with total sugar and total ginsenosides content measured in triplicate; results are expressed as mean ± SD. The resulting total sugar and total ginsenosides contents were 3.853 ± 0.025 mg/mL and 1.687 ± 0.002 mg/mL, respectively. Notably, endogenous glycosidases and proteases within the fermentation system formed a synergistic catalytic network. This network significantly enhanced the total sugar content by 2.048-fold and increased the total ginsenosides content to 1.448 times the pre-fermentation level (*p* < 0.05), primarily through the degradation of macromolecules such as polysaccharides and proteins present in the ginseng matrix. These results demonstrate the reliability and reproducibility of the fermentation process parameters optimized through orthogonal experimental design. This study successfully established a solid-state fermentation system with significant transformation efficiency, providing essential data support and a theoretical foundation for research on *A. oryzae*-ginseng solid-state fermentation. It also establishes a research basis for subsequent investigations into the transformation mechanisms and bioactivities.

**TABLE 3:** Total ginsenoside and total sugar contents in *Panax ginseng* under different conditions of the L_16_(4^3^) orthogonal array test.

Experiment number	A Fermentation time (h)	B Inoculum size (% v/v)	C Temperature (°C)	Total sugar content (mg/mL)	Total ginsenosides content (mg/mL)
1	1 (25 °C)	1 (1%)	1 (4 d)	1.111 ± 0.002	3.030 ± 0.005
2	1	2 (2.5%)	2 (6 d)	1.113 ± 0.001	2.865 ± 0.003
3	1	3 (5%)	3 (8 d)	1.120 ± 0.001	2.917 ± 0.057
4	1	4 (7.5%)	4 (10 d)	1.112 ± 0.001	3.058 ± 0.066
5	2 (28 °C)	1	3	1.225 ± 0.001	2.549 ± 0.002
6	2	2	4	1.335 ± 0.001	2.578 ± 0.003
7	2	3	1	1.123 ± 0.002	2.727 ± 0.002
8	2	4	2	1.125 ± 0.001	3.127 ± 0.002
9	3 (31 °C)	1	4	1.114 ± 0.001	3.277 ± 0.001
10	3	2	3	1.134 ± 0.001	3.178 ± 0.002
11	3	3	2	1.113 ± 0.002	3.161 ± 0.005
12	3	4	1	1.108 ± 0.001	2.563 ± 0.003
13	4 (34 °C)	1	2	1.110 ± 0.001	3.053 ± 0.001
14	4	2	1	1.113 ± 0.003	3.127 ± 0.003
15	4	3	4	1.115 ± 0.002	2.874 ± 0.003
16	4	4	3	1.110 ± 0.001	2.457 ± 0.006
K_1_	0.460	0.460	0.455		
K_2_	0.506	0.495	0.458		
K_3_	0.468	0.470	0.489		
K_4_	0.448	0.453	0.476		
k_1_	0.114	0.115	0.114		
k_2_	0.127	0.124	0.115		
k_3_	0.117	0.118	0.122		
k_4_	0.112	0.113	0.119		
*R*	0.015	0.0106	0.009		
Order of significance			A > B > C		
Optimal level	A2	B2	C3		
Optimal combination			A_2_B_2_C_3_		

### 3.3 Analysis of LC-MS

**FIGURE 1:**
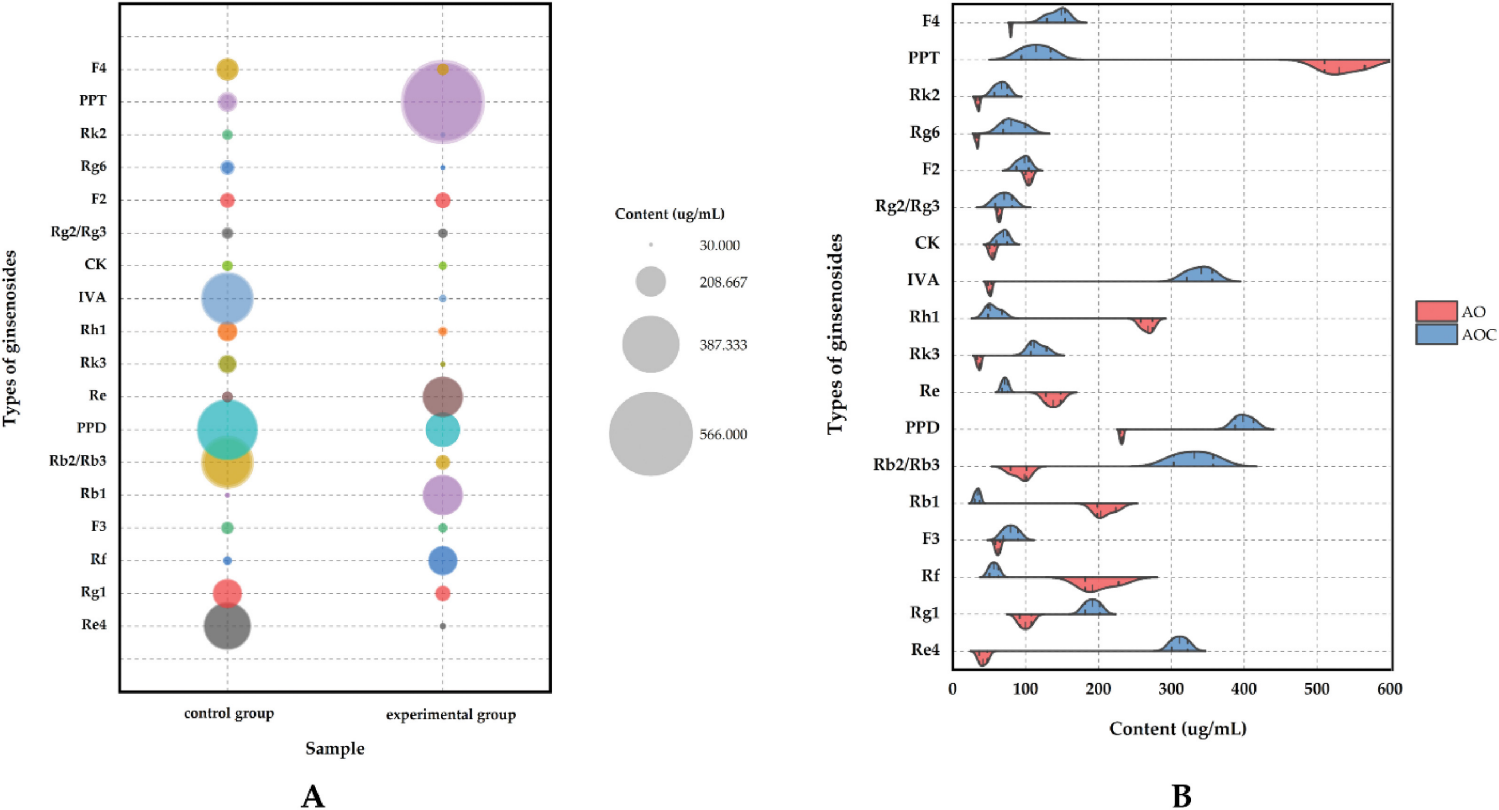
Changes in ginsenosides content before and after fermentation. **(A)** shows a bubble plot illustrating ginsenosides content changes pre- versus post-fermentation; **(B)** shows a bar chart comparing ginsenosides content changes pre- and post-fermentation.

Figure 1 depict the UPLC-MS/MS analysis (negative ion mode) of ginsenoside profiles in the control and *A. oryzae*-fermented ginseng groups. Figure 1 illustrates the quantitative changes in ginsenoside content before and after fermentation, while Supplementary Figure 2 shows the corresponding Base Peak Ion (BPI) chromatograms. Due to the complexity of the ginsenoside composition, which includes numerous isomers with similar polarities and closely eluting retention times, achieving complete chromatographic separation was challenging. To overcome this, a Waters ion mobility Q-TOF high-resolution mass spectrometry system was employed for enhanced characterization. Analysis of the negative ion mode data revealed 24 distinct peaks eluting between 10 and 50 min. By comparing these with reference standards, accurate molecular weights, MS/MS fragmentation patterns, and existing literature, a total of 18 ginsenosides were confidently identified. The detailed identification data for these 18 ginsenosides detected in the control and fermented groups are summarized in Table 4, respectively.

**TABLE 4:** Comparison of ginsenoside profiles between control and *A. oryzae*-fermented groups.

No.	Compound name	Molecular formula	[M-H]^–^ (m/z)	*A. oryzae*-fermented group		Control group	
				Retention (min)	Content (mg/g dry weight)	Retention (min)	Content (μ g/g dw dry weight)
1	Ginsenoside Re4	C_47_H_80_O_18_	977.5774	13.83	2.058 ± 0.020	13.15	15.573 ± 0.014
2	Ginsenoside Rg1	C_42_H_72_O_14_	845.5308	14.31	4.997 ± 0.075	14.31	9.585 ± 0.014
3	Ginsenoside Rf	C_42_H_72_O_14_	845.5308	20.64	10.007 ± 0.041	20.64	2.821 ± 0.010
4	Ginsenoside F3	C_41_H_70_O_13_	815.5116	23.81	3.093 ± 0.014	22.26	3.974 ± 0.013
5	Ginsenoside Rb1	C_54_H_92_O_23_	1,153.6542	24.19	10.427 ± 0.030	24.19	1.692 ± 0.011
6	Ginsenoside Rb2/Rb3	C_53_H_90_O_22_	1,123.6329	26.76	4.408 ± 0.071	26.76	16.668 ± 0.007
7	Ginsenoside PPD	C_56_H_94_O_24_	1,195.6570	28.15	11.594 ± 0.025	28.15	19.932 ± 0.012
8	Ginsenoside Re	C_48_H_82_O_18_	991.5839	30.70	6.883 ± 0.026	14.06	3.583 ± 0.010
9	Ginsenoside Rk2	C_42_H_70_O_12_	811.5128	33.51	1.756 ± 0.014	33.51	3.343 ± 0.011
10	Ginsenoside Rk3	C_36_H_60_O_8_	665.4519	34.14	1.805 ± 0.009	34.14	5.572 ± 0.013
11	Ginsenoside IVA	C_42_H_66_O_14_	793.4733	34.73	2.560 ± 0.037	34.73	17.055 ± 0.010
12	Ginsenoside Rh1	C_36_H_62_O_9_	636.4377	34.81	13.346 ± 0.053	34.81	2.786 ± 0.007
13	Ginsenoside CK	C_36_H_62_O_8_	665.4519	35.40	2.719 ± 0.018	35.40	3.488 ± 0.013
14	Ginsenoside Rg2/Rg3	C_42_H_72_O_13_	829.5285	35.71	3.176 ± 0.036	35.71	3.517 ± 0.021
15	Ginsenoside PPT	C_42_H_72_O_13_	829.5285	36.18	28.760 ± 0.042	36.18	5.718 ± 0.009
16	Ginsenoside F2	C_42_H_72_O_13_	829.5285	36.51	5.206 ± 0.039	36.51	4.922 ± 0.018
17	Ginsenoside Rg6	C_42_H_70_O_12_	811.5128	41.91	1.703 ± 0.017	41.91	4.015 ± 0.031
18	Ginsenoside F4	C_42_H_70_O_12_	811.5128	43.49	3.987 ± 0.061	43.49	7.456 ± 0.006

Mass spectrometric analysis suggested a targeted biotransformation within the protopanaxatriol-type ginsenosides, potentially following the pathway Rf → Rh1 → PPT. This process led to a substantial enrichment of these bioactive compounds, with the final products Rf and PPT increasing by 3.550-fold and 5.032-fold, respectively. The marked elevation of Rf, a characteristic marker for *P. ginseng*, is particularly noteworthy, as it underscores the potential of this fermentation process to enhance the signature bioactive profile of ginseng.

### 3.4 Protective effects of fermented ginseng extract on GES-1 cell viability against ethanol-induced injury

**FIGURE 2:**
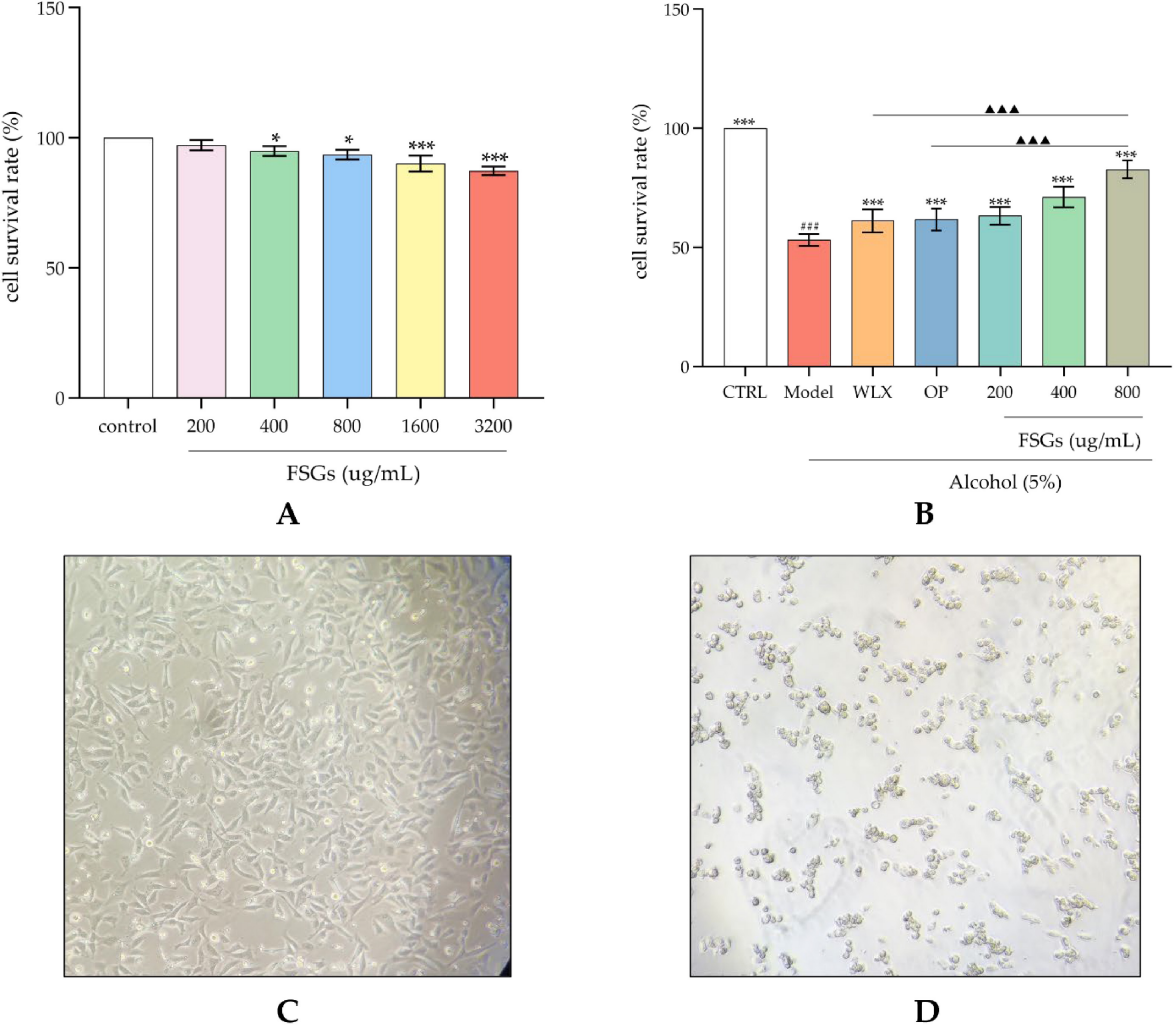
Effect of the fermentation extract on cell viability in ethanol-induced GES-1 cell injury. **(A)** shows the effect of ginsenosides extract on GES-1 cell viability; **(B)** shows the effect of ginsenosides extract on the viability of ethanol-induced damaged GES-1 cells. ###*p* < 0.001 vs. control group; ***p < 0.001 vs. model group; ▲▲▲*p* < 0.001 vs. positive control drug (indicating statistically significant differences). **(C)** shows micrographs of GES-1 cells under normal conditions; **(D)** shows micrographs of GES-1 cells in the ethanol-induced injury model state. *, ** and *** indicate statistical significance at different levels. *, **, and *** indicate statistical significance at different levels. The same applies to #, ##, ### and ▲, ▲▲, ▲▲▲.

As shown in Figure 2, exposure to ethanol significantly (*p* < 0.01) reduced the viability of GES-1 cells compared to the control. Treatment with the ginsenosides extract from fermented ginseng markedly attenuated this ethanol-induced injury in a dose-dependent manner, with all dose groups (low, medium, high) showing significant (*p* < 0.01) recovery in cell viability. Notably, the high-dose extract exhibited the most potent effect, surpassing even the positive control (*p* < 0.05).

### 3.5 Determination of anti-inflammatory and antioxidant levels in GES-1 cells by ELISA

**FIGURE 3:**
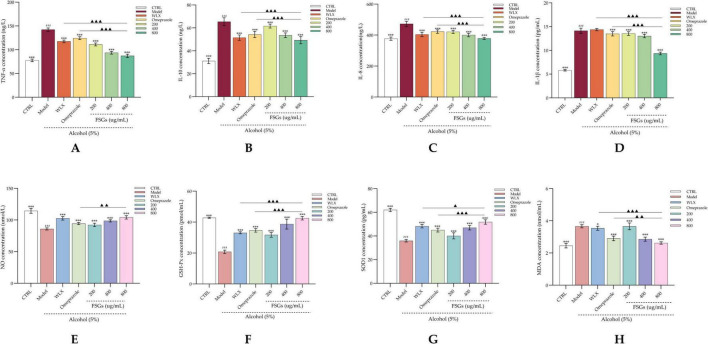
Effect of ginsenosides extract on antioxidant status in ethanol-induced GES-1 cells. **(A)** TNF-α content; **(B)** IL-10 content; **(C)** IL-8 content; **(D)** IL-1β content; **(E)** NO level; **(F)** GSH-Px activity; **(G)** SOD1 activity; **(H)** MDA level. ###*p* < 0.001 vs. control group; *p < 0.001 vs. model group; ▲*p* < 0.05, ▲▲*p* < 0.01, ▲▲▲*p* < 0.001 vs. positive control group (indicating statistically significant differences). *, ** and *** indicate statistical significance at different levels. *, **, and *** indicate statistical significance at different levels. The same applies to #, ##, ### and ▲, ▲▲, ▲▲▲.

As shown in Figures 3A–D, ethanol injury significantly elevated the levels of pro-inflammatory cytokines (IL-8, IL-1β, TNF-α) and the anti-inflammatory cytokine IL-10 in GES-1 cells compared to the control group (*p* < 0.01), confirming the successful induction of inflammation. Treatment with the ginsenosides extract significantly suppressed these inflammatory markers in a dose-dependent manner (*p* < 0.05), with the high-dose group showing the most pronounced reduction (*p* < 0.01). Notably, the anti-inflammatory effect of the high-dose extract surpassed that of the positive control (*p* < 0.01), whereas the low-dose group did not differ significantly from the model.

Similarly, ethanol exposure significantly decreased the activity of antioxidant enzymes (GSH-Px, SOD1) and NO levels relative to the control (*p* < 0.01; Figures 3E–H). The ginsenosides extract effectively restored these antioxidant markers in a dose-dependent fashion (*p* < 0.05), with the high-dose group again exhibiting the strongest effect (*p* < 0.01). Furthermore, the extract significantly attenuated ethanol-induced MDA accumulation, a marker of oxidative damage—with the high-dose group outperforming the positive control (*p* < 0.01). These results collectively demonstrate the potent anti-inflammatory and antioxidant activities of the fermented ginseng extract in protecting GES-1 cells from ethanol-induced injury.

### 3.6 Analysis of Western blot

**FIGURE 4:**
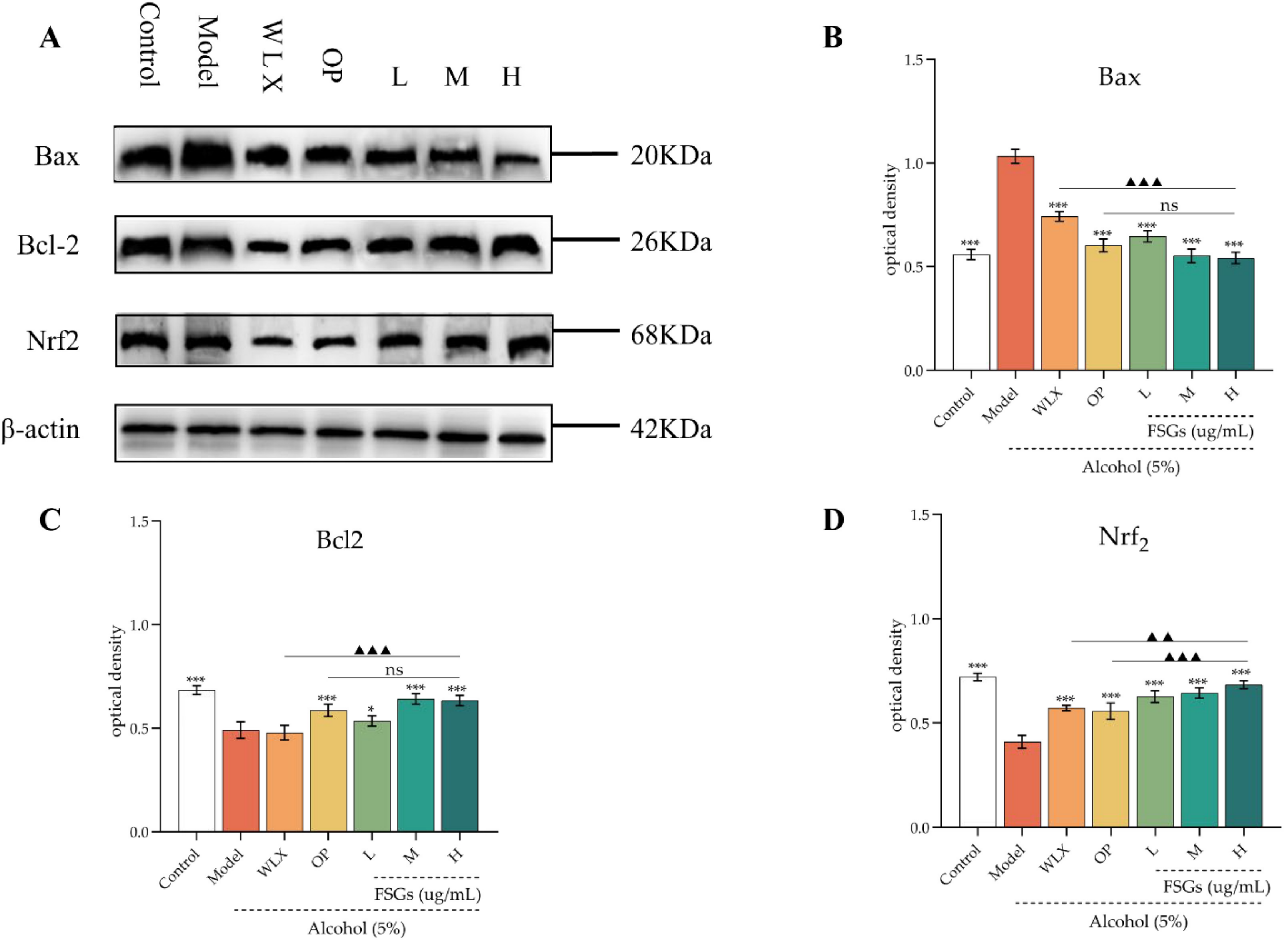
Effects of ginsenosides extract on Bax, Bcl-2, and Nrf_2_ expression in ethanol-damaged GES-1. **(A)** Representative Western blot; **(B–D)** Quantification of gray values for Bax, Bcl-2, and Nrf_2_. ###*p* < 0.001 vs. control; ***p < 0.001 vs. model; ▲▲*p* < 0.01, ▲▲▲*p* < 0.001 vs. positive control group (ns: not significant). *, ** and *** indicate statistical significance at different levels. *, **, and *** indicate statistical significance at different levels. The same applies to #, ##, ### and ▲, ▲▲, ▲▲▲.

As shown in Figure 4, ethanol-induced injury significantly upregulated the expression of the pro-apoptotic protein Bax and downregulated the anti-apoptotic protein Bcl-2 and the antioxidant regulator Nrf_2_ in GES-1 cells compared to the control group (*p* < 0.01). Treatment with the ginsenosides extract at low, medium, and high doses, as well as the positive control drug, significantly reversed these trends by downregulating Bax and upregulating Bcl-2 and Nrf_2_ (*p* < 0.05). Notably, the high-dose extract exerted more potent effects than the positive control, as reflected by a greater reduction in Bax and enhancement in Bcl-2 and Nrf_2_ expression (*p* < 0.05). The detrimental effects of ethanol on gastric mucosa involve multiple mechanisms beyond direct chemical corrosion, including the induction of apoptosis and necrosis (Yuan et al., 2021; Yang et al., 2020). In the present study, ethanol-treated GES-1 cells exhibited characteristic apoptotic morphological changes, such as cellular dehydration and chromatin condensation (Figures 2C,D), confirming the successful establishment of the injury model. Ethanol-induced gastric epithelial cell death is pathophysiologically linked to inflammatory responses and oxidative stress. The activation of inflammatory factors, together with the disruption of oxidative balance, synergistically exacerbates cellular damage. The NF-κB and MAPK signaling pathways are known to contribute to the production of inflammatory mediators such as NO, TNF-α, and IL-1 (Yang et al., 2020; Lawrence, 2009). The observed regulatory effects of the ginsenosides extract on apoptosis- and oxidative stress-related proteins suggest its potential involvement in these pathways. Further studies are warranted to elucidate the specific anti-inflammatory and antioxidant mechanisms underlying the protective effects of the *A. oryzae*-ginseng fermentation product.

## 4 Discussion

This study established and preliminarily optimized a food-grade, SSF process using *A. oryzae* for the targeted biotransformation of ginsenosides in *P. ginseng*. Unlike common fungi such as *A. niger*, which predominantly convert protopanaxadiol (PPD)-type ginsenosides, the present SSF process selectively remodeled the ginsenoside profile, leading to a significant enrichment of PPT-type derivatives. Quantitative analysis revealed that the levels of the marker ginsenoside Rf and its aglycone PPT increased by 3.55-fold and 5.03-fold, respectively (*p* < 0.05). This directed chemical transformation was directly linked to a functional enhancement: the resulting extract demonstrated significant, dose-dependent cytoprotective effects in an *in vitro* model of ethanol-induced injury in GES-1 gastric epithelial cells. The protection was evidenced by improved cell viability, a systematic downregulation of pro-inflammatory cytokines, enhanced activity of antioxidant enzymes, and a modulated balance of apoptosis-related proteins. These findings suggest that the fermentation-induced enrichment of PPT-type ginsenosides likely forms the chemical basis for the observed multi-target cytoprotective effect.

Process optimization identified fermentation time as a critical factor governing target ginsenoside accumulation, with content peaking around day 8 before declining. This dynamic profile likely reflects a stage-dependent balance between microbial growth, enzyme synthesis, and substrate availability. The initial accumulation phase may be associated with increased fungal biomass and the synergistic action of secreted hydrolytic enzymes (e.g., β-glucosidases, cellulases), which facilitate the release of ginsenosides from the matrix and initiate deglycosylation. The subsequent decline suggests that upon extended fermentation, the target products may undergo further conversion or degradation. Similar biphasic accumulation kinetics have been reported for secondary metabolite production in filamentous fungal fermentations, underscoring the necessity for precise control of the metabolic activity window (Wang et al., 2016, 2024; Daba et al., 2021).

Metabolite analysis supports a sequential deglycosylation pathway from Rf via Rh1 to PPT. Notably, in contrast to *A. niger*, which is frequently employed to transform PPD-type ginsenosides (e.g., conversion of Rb1 to Rg3), the *A. oryzae* strain used here exhibited a specific tendency to transform the sugar chain at the C-20 position of PPT-type ginsenosides (Wang et al., 2011; Chang et al., 2014). A pivotal step in this pathway involves the hydrolysis of an α-L-arabinopyranosidic bond, which is often considered a rate-limiting step in ginsenoside biotransformation due to the inherent stability of this glycosidic linkage (Poria et al., 2020). The efficient conversion of Rh1 to PPT observed in this study indicates that *A. oryzae* likely expresses a potent α-L-arabinosidase activity under the applied fermentation conditions. However, this inference remains primarily based on chemical phenotype. Future work employing functional studies such as proteomics, transcriptomics, and heterologous expression is required to identify and confirm the specific enzyme(s) responsible for this step, thereby fully elucidating the mechanism of this transformation pathway at the enzymatic and genetic levels (Shin and Oh, 2016).

While efficient, the microbial biotransformation strategy presents inherent limitations that pose significant challenges for practical application. Firstly, reliance on the intact microbial metabolic network, rather than a single purified enzyme, inevitably leads to complex product composition. Beyond the target rare ginsenosides, the fermentation system may contain non-target isomers, further degradation products, and microbial secondary metabolites. This complexity necessitates comprehensive safety assessments relying on systematic screening approaches like untargeted metabolomics. Secondly, the expression and activity of microbial enzyme systems are highly sensitive to environmental parameters (substrate, moisture, temperature). Fluctuations in these parameters, particularly in solid-state fermentation, can directly cause significant batch-to-batch variability, presenting a major challenge for process standardization and reproducibility. Finally, scaling up from laboratory to industrial scale presents non-linear engineering hurdles. Heat and mass transfer gradients, unavoidable in large-scale reactors, can alter microbial physiology and metabolic consistency, potentially affecting the homogeneity and quality of the final product. Furthermore, even when using a species with a history of safe use like *A. oryzae* (generally recognized as safe, GRAS), its application for a new substrate to produce a new component is typically viewed as a “new use” by regulators. Consequently, regulatory focus will center on providing strain-specific evidence of genetic stability, absence of toxin production, and a complete safety dossier for the final product, constituting a substantial regulatory threshold prior to market approval. Therefore, advancing this technology toward application requires simultaneously addressing a series of interconnected scientific and engineering issues, including control of product complexity, enhancement of process robustness, validation during scale-up, and establishment of regulatory compliance.

In conclusion, this study demonstrates that directed SSF using food-grade *A. oryzae* can effectively steer the ginsenoside profile of ginseng toward the more bioactive PPT-type and yield an extract with clear *in vitro* gastroprotective potential. This work provides a novel and safe biomanufacturing route for developing higher-value ginseng products. To translate this promise into reality, subsequent research should follow a stepwise path: (1) Mechanistic investigation: Employ multi-omics integrated with *in vitro* enzymology to identify and characterize the key enzyme(s) responsible for α-L-arabinosidic bond hydrolysis; (2) Integrated process and safety validation: Conduct process verification in scaled-up equipment alongside comprehensive safety monitoring using untargeted metabolomics; (3) Efficacy and safety substantiation: Evaluate the overall protective efficacy against gastric mucosal injury in relevant animal models and complete standardized toxicological evaluations. Addressing these technical, analytical, and regulatory requirements will be essential for industrial development and commercialization.

## 5 Conclusion

This study established and preliminarily optimized a solid-state fermentation system using *A. oryzae* for targeted biotransformation of ginseng under the reported conditions. UPLC-QTOF-MS/MS profiling revealed enrichment of several high-value rare ginsenosides, with evidence consistent with conversion of the ginseng marker Rf toward protopanaxatriol (PPT). The fermented extract showed enhanced bioactivity *in vitro* and no observable cytotoxicity in GES-1 cells within the tested concentration range. These findings support the potential of *A. oryzae*-mediated SSF as a route to produce higher-value ginseng ingredients. However, further work is required to validate the responsible enzymatic mechanism, to perform comprehensive safety and mycotoxin screening, and to confirm efficacy and safety in appropriate animal models before application as a nutraceutical.

## Data Availability

The original contributions presented in this study are included in this article/Supplementary material, further inquiries can be directed to the corresponding authors.
