# Supplementary material for: Aspergillus oryzae solid-state fermentation enriches protopanaxatriol-type ginsenosides in Panax ginseng and confers cytoprotective effects in vitro
Source: Front Microbiol. 2026 Mar 6;17:1747324. doi: 10.3389/fmicb.2026.1747324 (PMC13002569; doi:10.3389/fmicb.2026.1747324)
Supplement: Supplementary file 1 [file Data_Sheet_1.docx]

Supplementary Material

# Supplementary Tables

**Supplementary Table S1.** Gradient elution program for mobile phase composition

| Time  (min) | A%  (0.1% formic acid in water) | B%  (0.1% formic acid in acetonitrile) |
| --- | --- | --- |
| 0 | 85 | 15 |
| 7 | 80 | 20 |
| 13 | 70 | 30 |
| 23 | 65 | 35 |
| 27 | 62 | 38 |
| 31 | 55 | 45 |
| 35 | 50 | 50 |
| 40 | 45 | 55 |
| 45 | 42 | 58 |
| 50 | 60 | 40 |
| 60 | 90 | 10 |

# Supplementary Figures


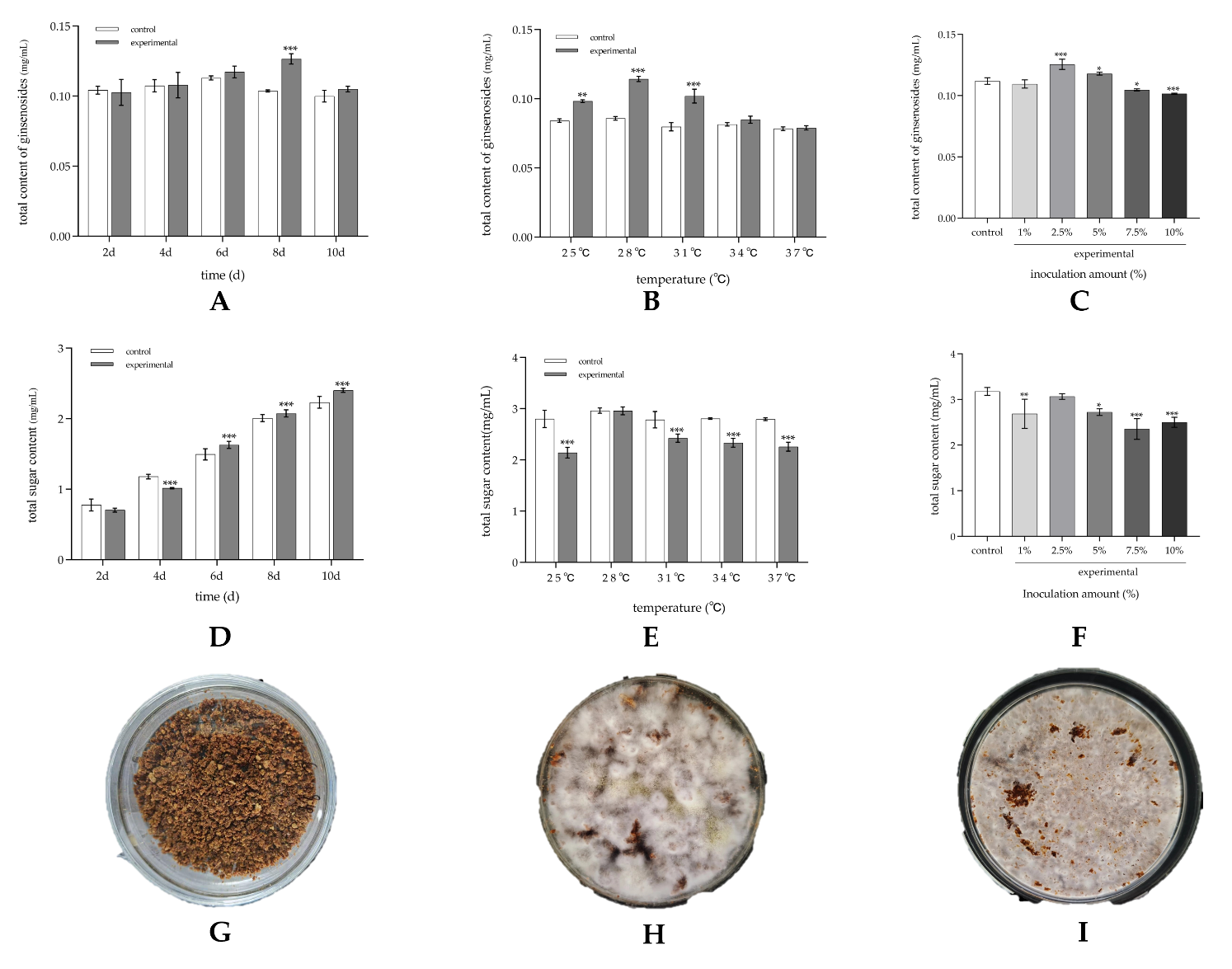


**Supplementary Figure S1.** Single-factor test results of *A. oryzae*-ginseng solid-state fermentation. **(A)**, **(B)**, **(C)** shows the effects of fermentation time, temperature, and inoculum size on total ginsenosides content; **(D)**, **(E)**, **(F)** shows the effects of fermentation time, temperature, and inoculum size on total sugar content; asterisks denote statistically significant differences compared to the control group (* *p* < 0.05, ** *p* < 0.01, *** *p* < 0.001). **(G)** shows the top view of the control group in the single-factor test; **(H)** shows the top view of the experimental group in the single-factor test; **(I)** shows the bottom view of the experimental group in the single-factor test.


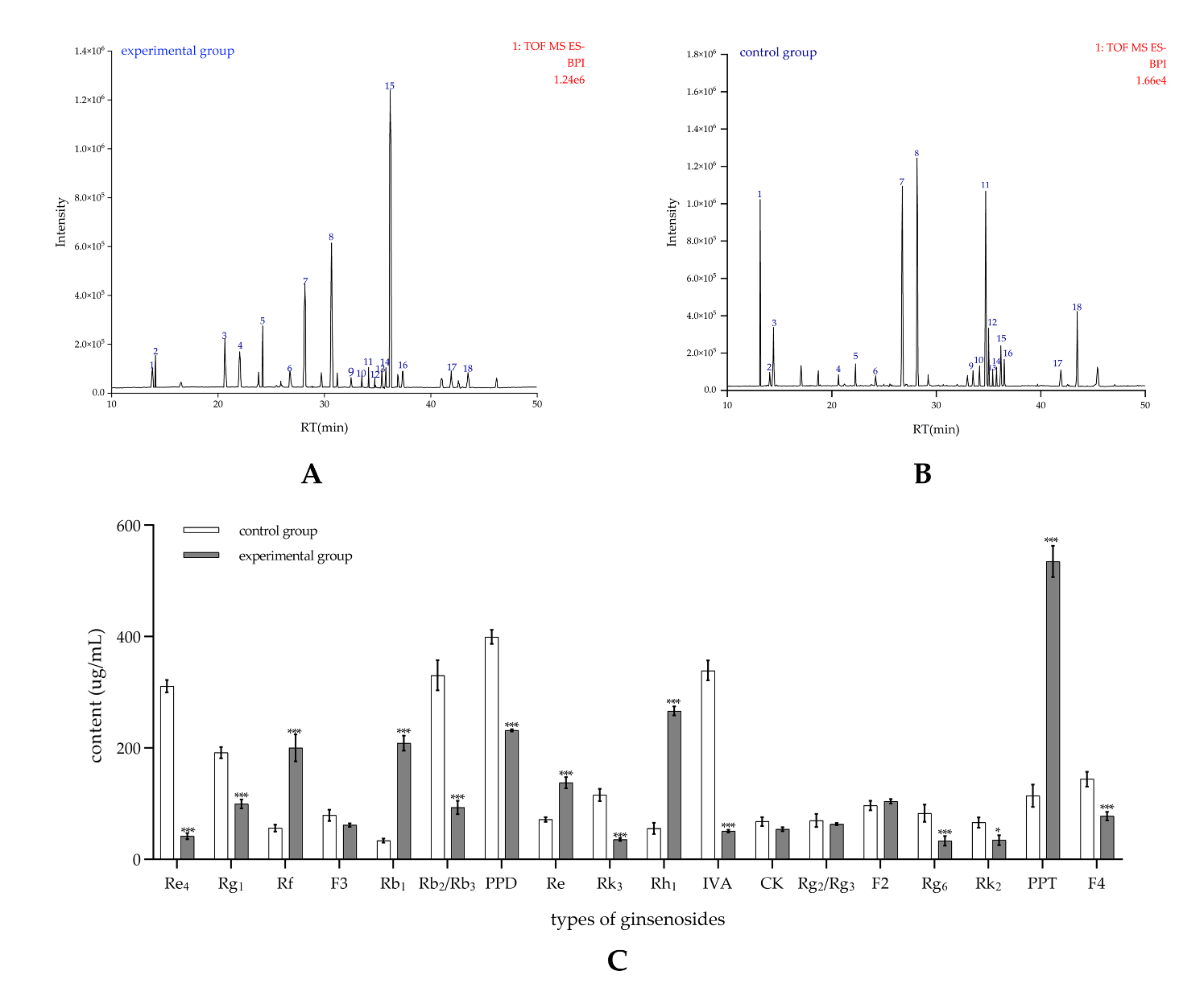


**Supplementary Figure S2.** Changes in ginsenosides content before and after fermentation. **(A)** shows the total ion chromatogram (TIC) of the experimental group; **(B)** shows the TIC of the control group; **(C)** shows the quantitative comparison of ginsenosides levels in the form of a bar chart for the two groups. Asterisks denote statistically significant differences compared to control group: * *p* < 0.05, *** *p* < 0.001.

# Supplementary Western Blot Figures

**
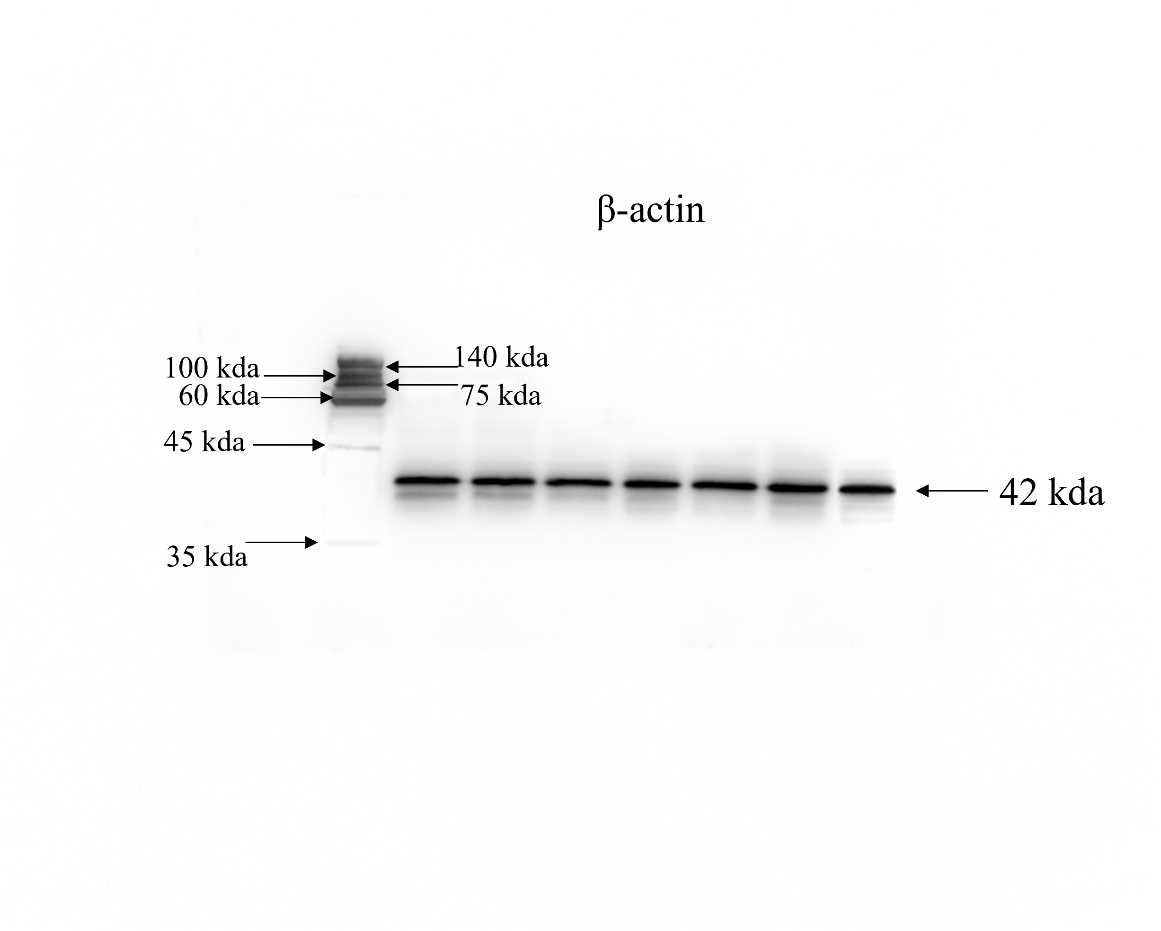
**

**Supplementary Figure S3.** Uncropped Western blot image of β-actin (loading control) showing molecular weight markers and the protein band. Lanes from left to right: Control, Model, Weile Xin (WLX), Omeprazole, Low-dose (L), Medium-dose (M), High-dose groups (H), corresponding to the order in the manuscript.


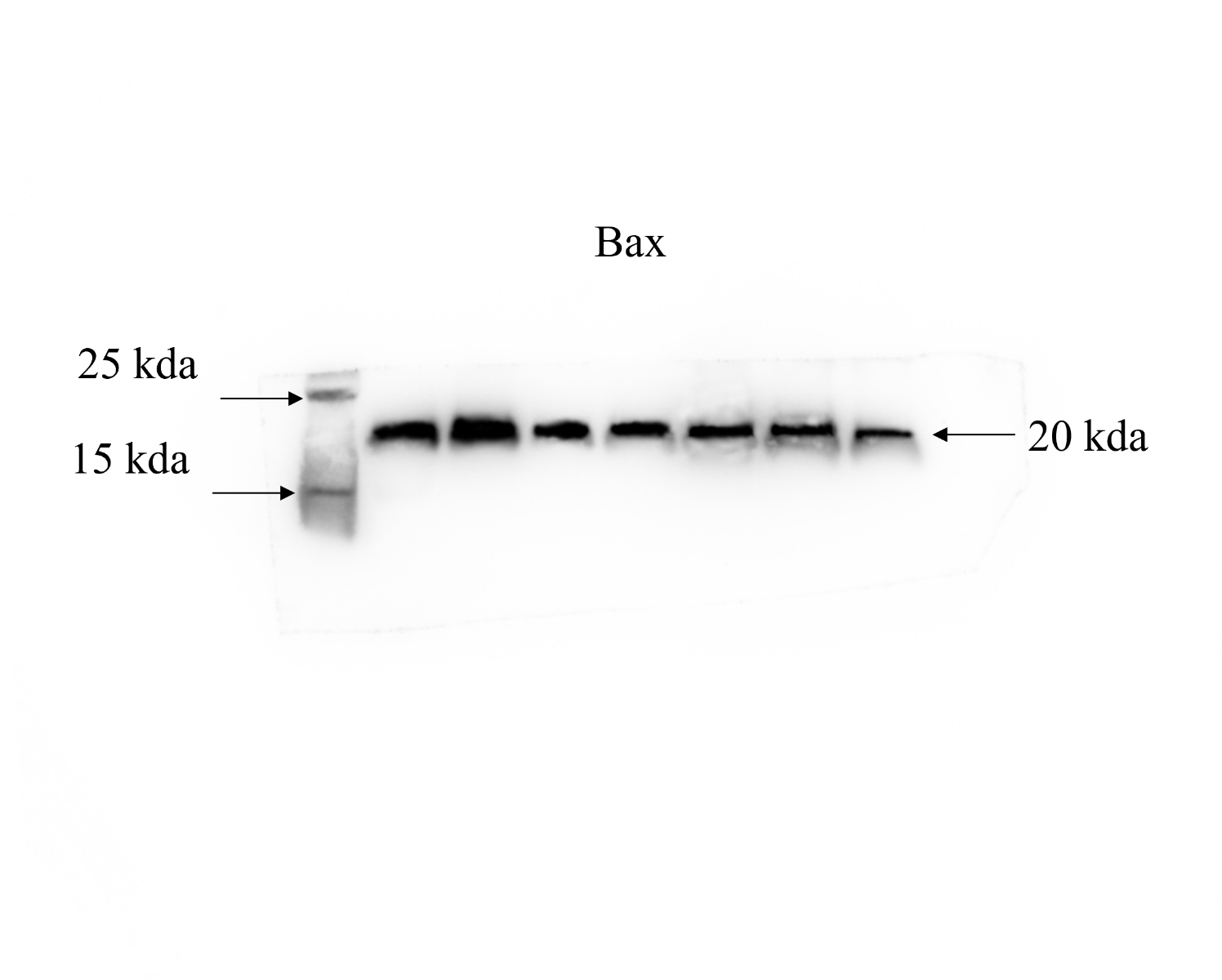


**Supplementary Figure S4.** Uncropped Western blot image of Bax, showing molecular weight markers and the specific protein band. Lanes from left to right are: Control, Model, Weilexin (WLX), Omeprazole, Low-dose (L), Medium-dose (M), and High-dose (H) groups, corresponding to the order in the manuscript.


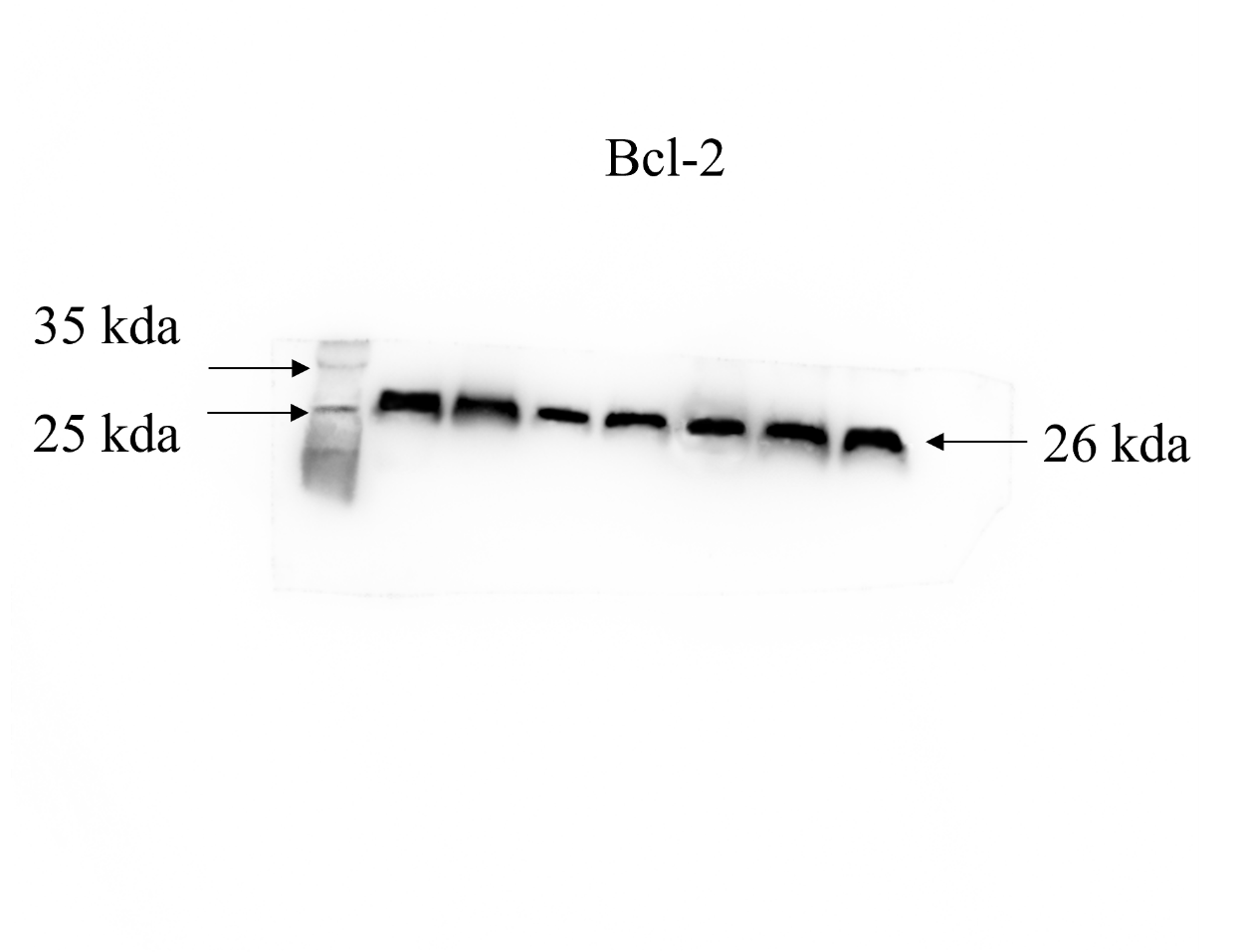


**Supplementary Figure S5.** Uncropped Western blot image of Bcl-2, showing molecular weight markers and the specific protein band. Lanes from left to right are: Control, Model, Weilexin (WLX), Omeprazole, Low-dose (L), Medium-dose (M), and High-dose (H) groups, corresponding to the order in the manuscript.


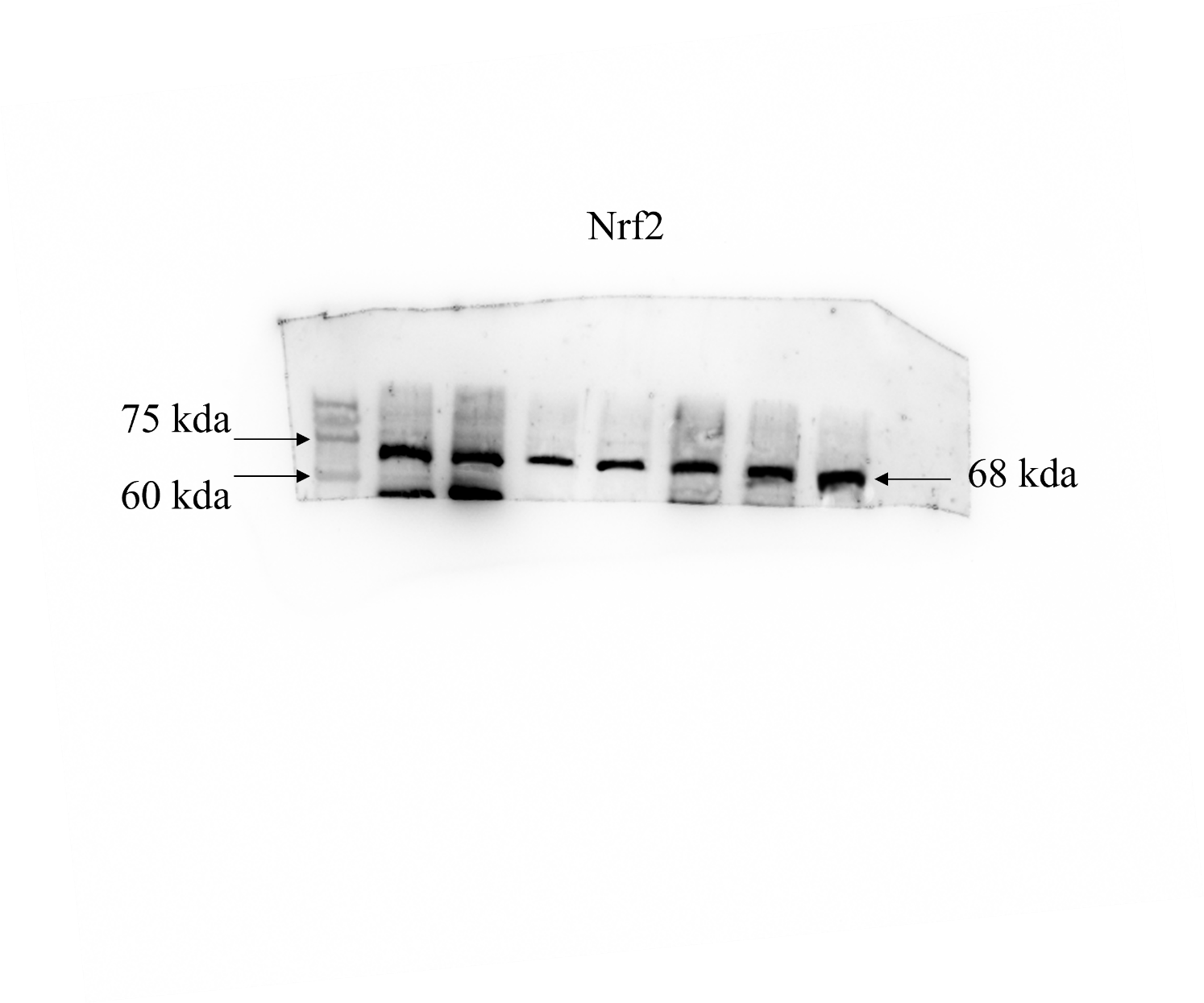


**Supplementary Figure S6.** Uncropped Western blot image of Nrf_2_, showing molecular weight markers and the specific protein band. Lanes from left to right are: Control, Model, Weilexin (WLX), Omeprazole, Low-dose (L), Medium-dose (M), and High-dose (H) groups, corresponding to the order in the manuscript.
