# Supplementary figures and images for: Aspergillus oryzae solid-state fermentation enriches protopanaxatriol-type ginsenosides in Panax ginseng and confers cytoprotective effects in vitro
Source: Front Microbiol. 2026 Mar 6;17:1747324. doi: 10.3389/fmicb.2026.1747324 (PMC13002569; doi:10.3389/fmicb.2026.1747324)

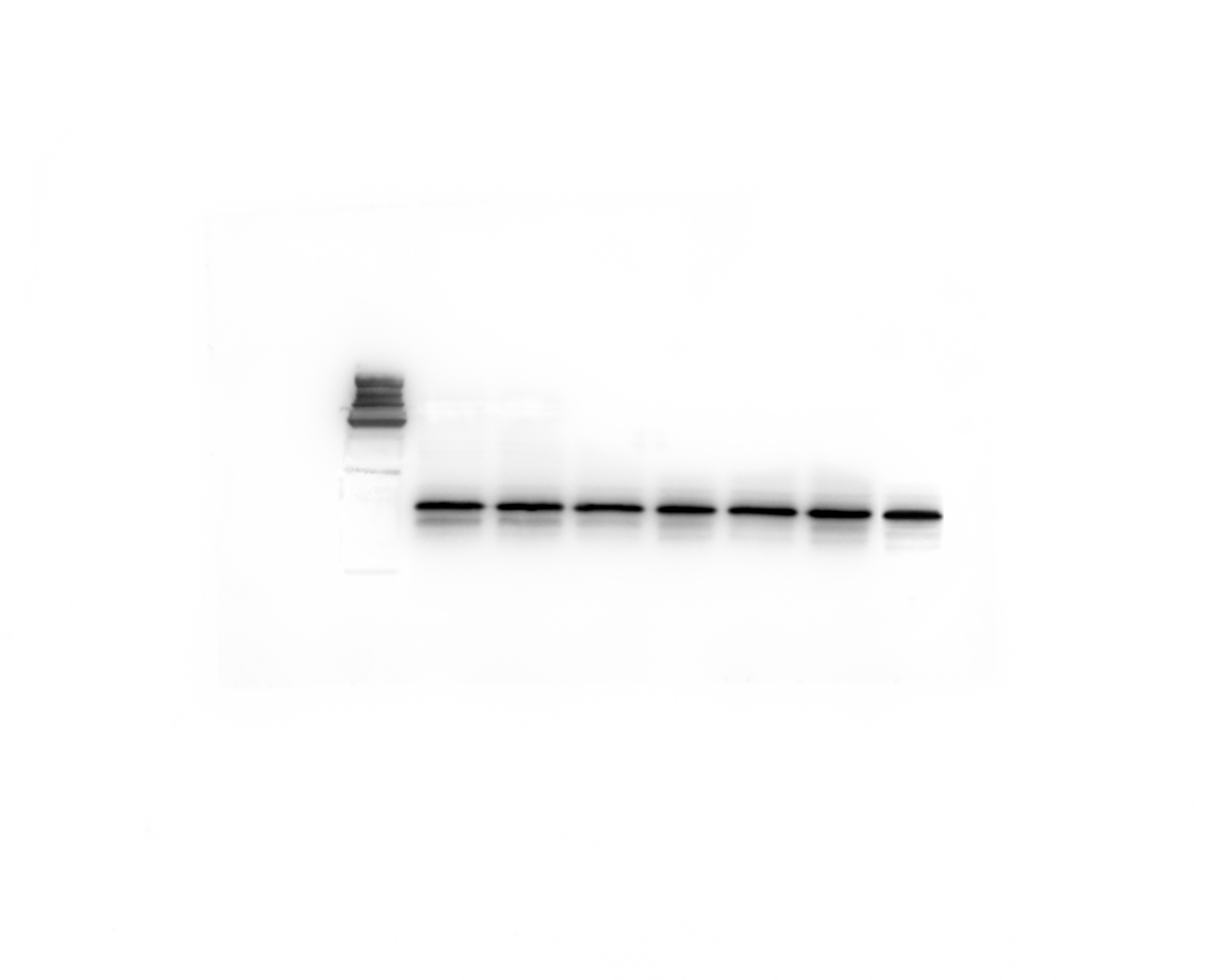

Supplement: Supplementary file 2 [file Image_1.tif]

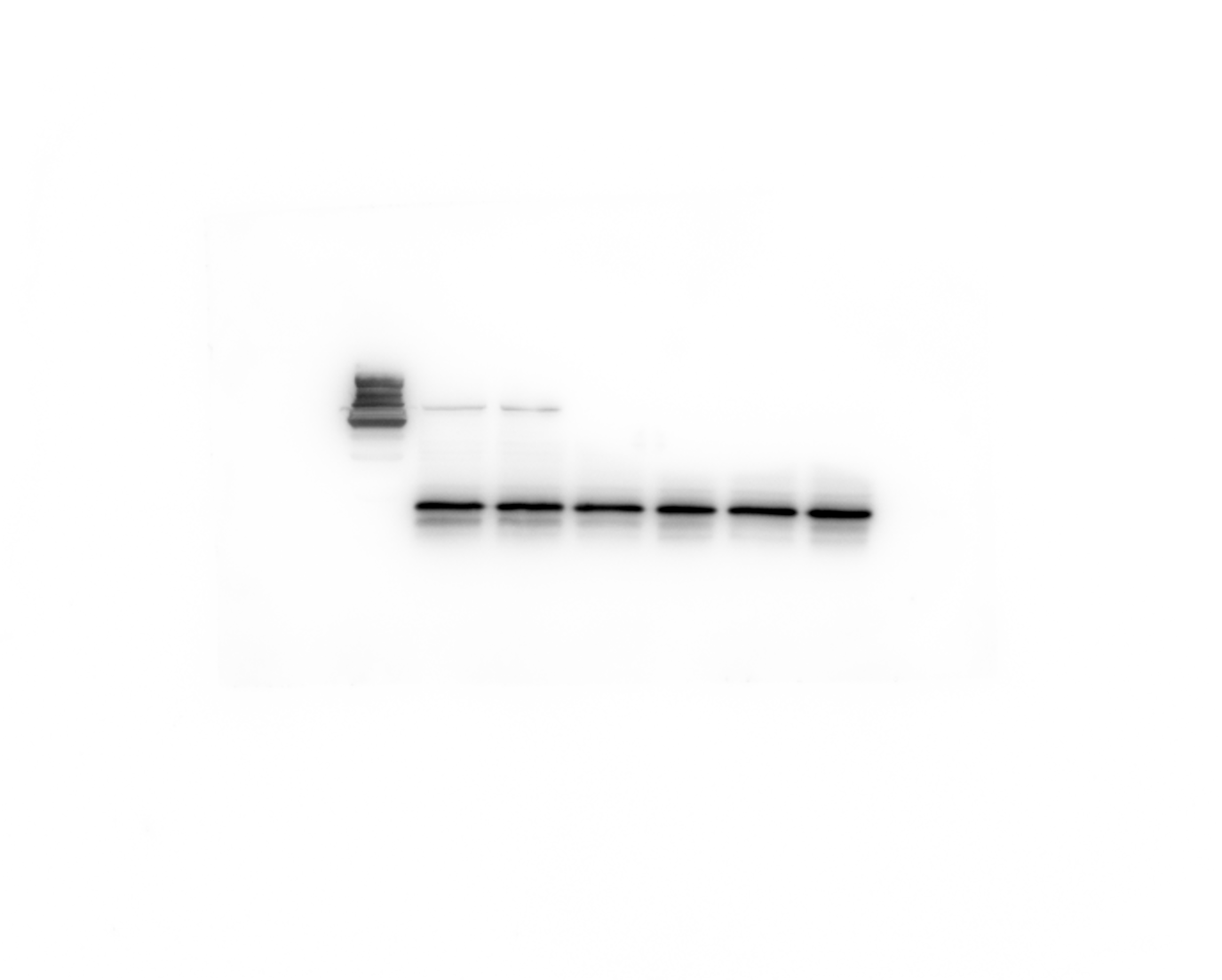

Supplement: Supplementary file 3 [file Image_2.tif]

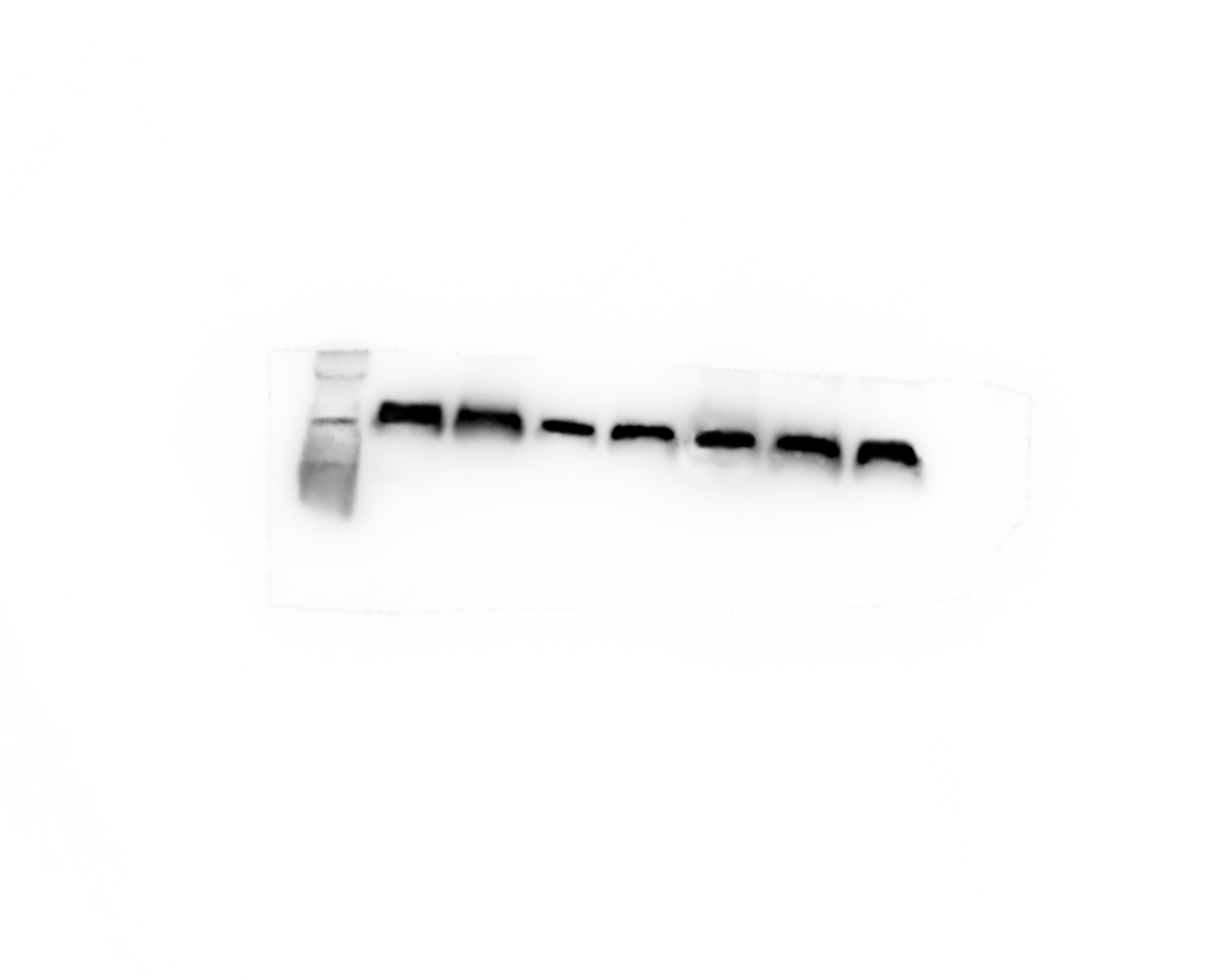

Supplement: Supplementary file 4 [file Image_3.tif]

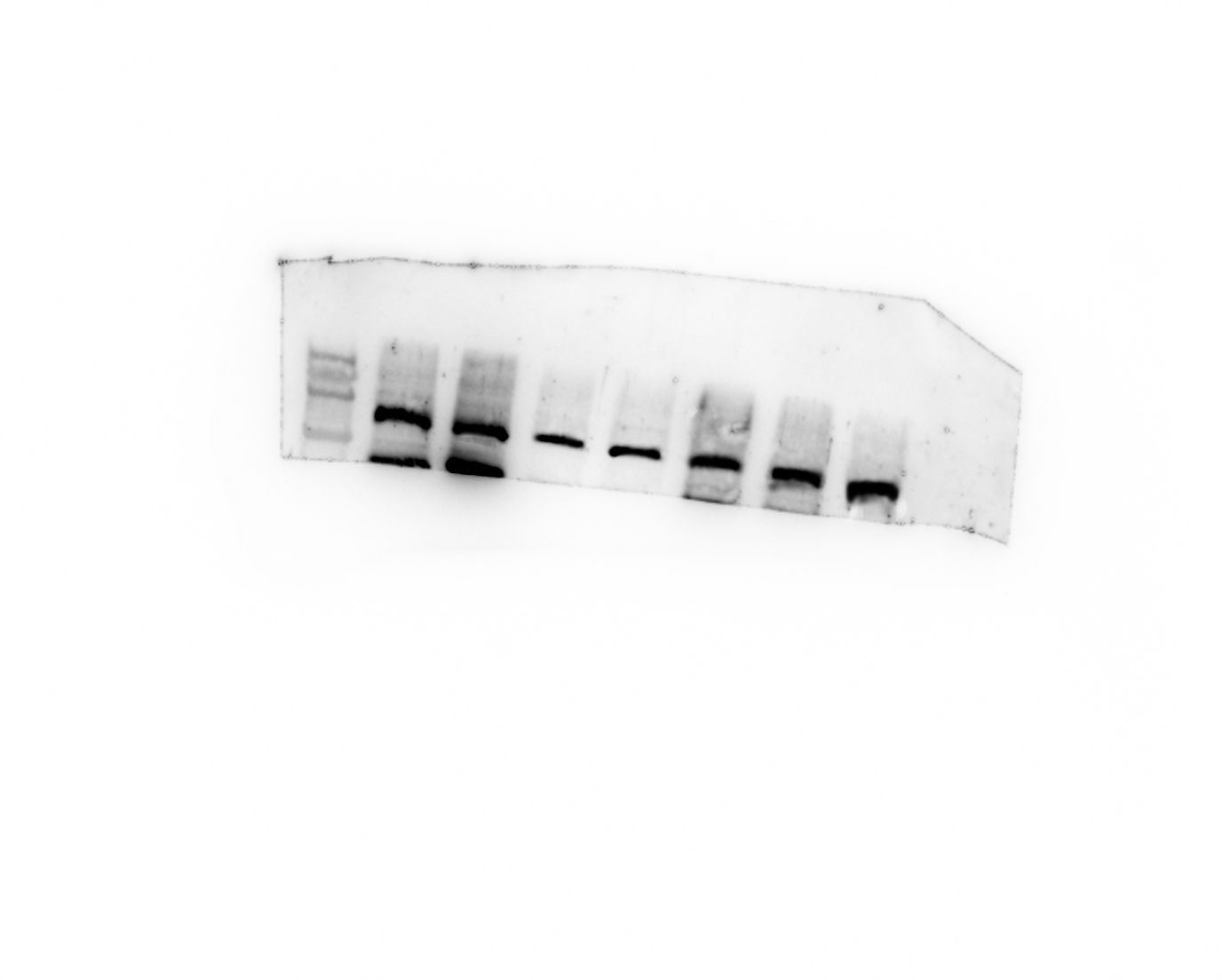

Supplement: Supplementary file 5 [file Image_4.tif]
